# Experiences of community pharmacists involved in the delivery of a specialist asthma service in Australia

**DOI:** 10.1186/1472-6963-12-164

**Published:** 2012-06-18

**Authors:** Lynne M Emmerton, Lorraine Smith, Kate S LeMay, Ines Krass, Bandana Saini, Sinthia Z Bosnic-Anticevich, Helen K Reddel, Deborah L Burton, Kay Stewart, Carol L Armour

**Affiliations:** 1 School of Pharmacy, Curtin Health Innovation Research Institute, Curtin University, Perth, Australia; 2 School of Pharmacy, The University of Queensland, Brisbane, Australia; 3 Faculty of Pharmacy, The University of Sydney, Sydney, Australia; 4 Woolcock Institute of Medical Research and Faculty of Medicine, The University of Sydney, Sydney, Australia; 5 School of Medical and Applied Sciences, Central Queensland University, Rockhampton, Australia; 6 School of Biomedical Sciences, Charles Sturt University, Wagga Wagga, Australia; 7 Centre for Medicine Use and Safety, Monash University, Melbourne, Australia

**Keywords:** Pharmacy, Asthma, Disease management service, Experiences, Feedback

## Abstract

**Background:**

The role of community pharmacists in disease state management has been mooted for some years. Despite a number of trials of disease state management services, there is scant literature into the engagement of, and with, pharmacists in such trials. This paper reports pharmacists’ feedback as providers of a Pharmacy Asthma Management Service (PAMS), a trial coordinated across four academic research centres in Australia in 2009. We also propose recommendations for optimal involvement of pharmacists in academic research.

**Methods:**

Feedback about the pharmacists’ experiences was sought via their participation in either a focus group or telephone interview (for those unable to attend their scheduled focus group) at one of three time points. A semi-structured interview guide focused discussion on the pharmacists’ training to provide the asthma service, their interactions with health professionals and patients as per the service protocol, and the future for this type of service. Focus groups were facilitated by two researchers, and the individual interviews were shared between three researchers, with data transcribed verbatim and analysed manually.

**Results:**

Of 93 pharmacists who provided the PAMS, 25 were involved in a focus group and seven via telephone interview. All pharmacists approached agreed to provide feedback. In general, the pharmacists engaged with both the service and research components, and embraced their roles as innovators in the trial of a new service. Some experienced challenges in the recruitment of patients into the service and the amount of research-related documentation, and collaborative patient-centred relationships with GPs require further attention. Specific service components, such as the spirometry, were well received by the pharmacists and their patients. Professional rewards included satisfaction from their enhanced practice, and pharmacists largely envisaged a future for the service.

**Conclusions:**

The PAMS provided pharmacists an opportunity to become involved in an innovative service delivery model, supported by the researchers, yet trained and empowered to implement the clinical service throughout the trial period and beyond. The balance between support and independence appeared crucial in the pharmacists’ engagement with the trial. Their feedback was overwhelmingly positive, while useful suggestions were identified for future academic trials.

## Background

For some decades, there has been a recognised, and increasing, need for community pharmacists to demonstrate their expertise in healthcare delivery, including, but no longer limited to, medication management. This role uniquely positions pharmacists as intermediaries between patients and prescribers, yet practising independently from prescribers, with roles incorporating medication review, case identification and disease monitoring, patient education and advocacy, and referral to prescribers and other health professionals.

Roles for community pharmacists as medication and disease state management experts have been reflected in the development of specialist services delivered within pharmacies. Patients with chronic medical conditions are particularly well suited to receive such services from pharmacists, due to their need for ongoing monitoring and management of (often) multiple medications. To this end, services based on the management of asthma [1-7], diabetes [8,9], hypertension [10] and bodyweight [11] have been tested, with positive clinical [1,3,5-9] and economic [4,6,7,9] impact data.

The successful establishment of new services requires rigorous programs of evaluation. Pharmacists wishing to develop expertise in chronic disease management, and subsequently deliver a useful, sustainable, economically viable and professionally rewarding service, have been encouraged to participate in academic trials of specialist services. The researchers commonly provide advanced clinical education, skills training, practical support to establish and deliver the service, and feedback on service standards and patient outcomes. Participating pharmacists also stand to benefit from peer support of colleagues and other health care professionals who have been informed of the trial and, in funded projects, potentially receive financial compensation for their time commitment.

Previously, we have investigated the motivation for pharmacists to participate in research trials; [12] however, uptake of professional services requires commitment beyond the period of the research program. Reports from completed trials suggest that pharmacists may find the implementation and delivery of a specialist service a significant leap beyond their everyday practice. [10,13,14]

Consistent with the theory on the diffusion of innovation [14,15], the pharmacists accepting the challenge of trialling a new cognitive service may be considered “innovators” and/or “early adopters”. However, the profession needs to move beyond this early phase if services are to be sustainable and accepted by both patients and pharmacists in practice. Research promoting the implementation of professional services has provided guidance on human resources, communication and teamwork, and external support for the service [14], while reports following the implementation of services often focus on the patient outcomes, rather than the pharmacists’ experiences as providers of a service [16]. Despite the emergence of a number of specialist disease-management services in pharmacies, there has been little published research to explore pharmacists’ experiences as pioneers of a structured disease state management service, in balancing the additional workload involved in service provision, learning and applying new skills, managing documentation requirements, marketing themselves in a different context, and in developing new relationships [13,17]. Internationally, Canadian researchers have reported common challenges in pharmacists’ engagement with disease state management services, including time constraints, access to information, formation of working relationships and absence of a practice model [18]. Whether these are universal depends on the program under study and constraints of each country’s healthcare system.

Thus, it is important to explore the barriers and facilitators from the perspective of the service provider, the pharmacist. This paper reports the exploration of pharmacists’ experiences as service providers in an implementation trial of a novel patient-focused specialist asthma service (the Pharmacy Asthma Management Service, PAMS) in Australia [3]. In addition, we sought their views on how their patients received the service, the pharmacists’ interactions with other health care professionals (particularly general practitioners), as a result of the asthma service, the impact of the service on their role, their opinions on offering such a service in future, and feedback on the support from the investigators to guide the service implementation.

The objective of this study was to gain feedback from the participating pharmacists on their experiences as providers of the specialist asthma service. A second objective, based on the results of these data, was to propose recommendations for optimal involvement of pharmacists in a multi-centre academic research trial of this nature. Based on the researchers’ experience with the original randomised controlled trial of this service [5] and a similar service for diabetes management by pharmacists in Australia, [13] it was hypothesised that the model for our trial, offering support, guidance and motivation for the participating pharmacists, would generate mostly positive experiences amongst the pharmacists and would facilitate constructive feedback about the research protocol from the participants to the researchers.

## Methods

This implementation trial was conducted as a cluster randomised controlled study comparing the original service model [5] of four patient visits in six months with a less-intensive model of three visits in six months. Recruitment of the service providers was undertaken geographically by pharmacy, with proportional representation of the number of community pharmacies in regional and metropolitan areas in four States/Territories in Australia (New South Wales, Victoria, Queensland and the Australian Capital Territory). The recruitment was managed online by The Pharmacy Guild of Australia, as part of the research funding agreement. Pharmacy eligibility criteria were the availability of one pharmacist to attend training for credentialing as the service provider, a private consultation space, and availability of a second pharmacist or time outside normal hours to provide uninterrupted appointments for the service. The pharmacists received advanced training in asthma management over two days at each research centre; prior to this, they all undertook self-directed study using provided reading materials. Training incorporated risk assessment, pathophysiology of asthma, asthma medications, current treatment guidelines, [19] patient education, goal setting, adherence assessment, and spirometry. The training concluded with an examination of both knowledge and skills, with successful completion awarded accreditation by the Australian Association of Consultant Pharmacy. Following training, a total of 106 pharmacists were authorised to commence the asthma management service, and 93 proceeded to provide the service.

Key features of this pharmacy asthma service were derived from the original randomised control trial of the service [5]. Each pharmacist recruited up to 10 regular customers of their pharmacy who were identified as being at risk of poor asthma outcomes, either by having sub-optimally-controlled asthma or no asthma review in the previous six months [2]. Pharmacists had two to three months to complete recruitment, and progress was monitored and encouragement provided approximately three weeks after the training. A median of five patients per pharmacy completed the service (range 0–10). Patients attended either three or four visits at their pharmacy over a six-month period in 2009 where spirometry and asthma control were measured, with the consultations focusing on asthma control and factors influencing asthma control, smoking cessation (where warranted), medication use, action plan ownership, inhaler technique and goal setting. The initial patient consultation required completion of several validated questionnaires by the patient, with further questionnaires at subsequent visits (partial data were included in the case of patients withdrawing from the service). Further, the pharmacists maintained research-related documentation, including records of their clinical interventions, patients’ spirometry readings, and communication with patients and other healthcare providers. General practitioners (GPs) in the area surrounding each study pharmacy were sent information about the study by the investigators, and the pharmacists were encouraged to contact, and preferably meet with, their local GPs to explain the service. Pharmacists were trained in mechanisms to refer patients to their GP for issues such as medication changes, suboptimal spirometry values and provision of written action plan; a template for referral and an asthma action plan was provided within the patient file.

The study was conducted in accordance with the Helsinki Declaration, and the study protocol was approved by the Human Research Ethics Committee of The University of Sydney (11-2008/11419), with secondary approvals from the Medical Research Ethics Committee of The University of Queensland, the Human Research Ethics Committee of Charles Sturt University, and the Monash University Human Research Ethics Committee. All pharmacists and patients provided written informed consent for their participation. Pharmacists were not compensated for their time in providing reflective data, although their time spent with study patients was remunerated via payments approved in the research protocol.

Feedback was sought from participating pharmacists at three time points (Table 1), to facilitate reflection as the project progressed. The data were obtained using focus groups, supplemented by individual interviews for those unavailable for their scheduled focus group. Pharmacists were sampled randomly from rural and urban pharmacies, and individuals participated at only one time point, to minimise their research load. The aim was to recruit six to eight pharmacists for each focus group. Focus groups of this size have been established as optimal to encourage contribution of each participant and to compare and contrast their experiences [20].

**Table 1:** Pharmacist feedback protocol

Timepoint	Research Centre(s) Represented	Method	Number of Pharmacist Participants (n = 32)
Following completion of at least one patient’s initial consultation (“Early”)	All	Focus group	7
End of service delivery (6 months; 3 or 4 consultations per patient) (“Completion”)	Victoria	Focus group	12*
		Individual interviews	6
6 months following completion of service delivery (“Follow-up”)	All	Focus group	6
		Individual interview	1

*Significant non-contact rate by pharmacists registered for the focus group.

Focus groups and interviews were conducted via telephone after business hours, for the convenience of participants and to facilitate interaction in the focus groups between geographically distanced pharmacists. A semi-structured interview guide, based on previous research experience, was developed (Additional file 1), with questions centred around pharmacists’ views on their training for the service, their perceptions regarding their patients’ experience of receiving the service, interactions with health professionals, and the future for a service model such as this. All focus groups were facilitated by two investigators from the main research centre. The individual interviews were shared between three interviewers from the main research centre, who were trained in a consistent approach. In cases where the interviewer/facilitator was not known to the pharmacist, rapport was established prior to the discussion via introduction to the researcher and general conversation about pharmacy practice, and an overview of how the focus group discussions would unfold.

Interviews and focus group discussions were transcribed verbatim by professional transcribers independent to the research team. The transcripts were analysed manually for the identification of underlying concepts by one researcher in accordance with the semi-structured focus group guide, with consensus then achieved by two more investigators for interpretation of the content. Comments were extracted as both confirmative and disconfirmative evidence to explore the breadth of responses and pharmacists’ experiences.

## Results

All pharmacists approached to give feedback agreed; however, in a small number of cases, the pharmacist could not commit to attending the focus group. Overall, 32 pharmacists were involved in either a focus group or individual interview (Table 1): seven following completion of at least one patient’s initial consultation (“Early”, quotations annotated “FG1”), 18 after completion of the service (“End”, quotations annotated “FG2” and “I2”), and seven six months after service completion (“Follow-up”, quotations annotated “FG3” and “I3”). Eight concepts relating to the service provision were identified from the transcript analysis: the training, challenges with the recruitment and consultation of patients, the service components, logistics of the service provision, relationships with the GPs, perceptions of the patients’ experiences, professional rewards, and the future of the service.

### **The training**

The clinical and practical skills training in asthma management was perceived to train pharmacists to a high standard:

"*“My husband’s a GP, and he looked through the course and what we learnt, and he said we’re better qualified than most GPs now to handle asthma.” (FG3-1)*"

Training in spirometry was described by one participant as *“probably the pinnacle part that you want to take away from that day”* (FG1-4), and there were requests for greater emphasis on this section to improve pharmacists’ confidence, exemplified by the pharmacist who was *“dead scared a doctor would ask me, ‘what does this [spirometry result] mean?’”* (FG3-2)

Comments suggested that training group sizes should be limited to 20 people, as per the size of the groups in this study, for optimal skills development and peer support.

### **Challenges with recruitment of and consultation with patients**

Confidence with recruiting patients was critical to the patients’ engagement with the service; some pharmacists found promoting the service daunting and a new experience, and some requested more training in patient recruitment into this type of clinical service. Key recruitment strategies were revealed:

"*“Work out what are the selling points … and why is it good for them to be involved in a university trial, and what outcomes we’re after … and how to hit their trigger points when you’re recruiting them.” (FG1-6)*"

"*“You need to talk to patients at length and allay their fears and talk about their desires and all that sort of stuff … the time to do this is in the afternoon when you’ve got the time.” (FG2-N3)*"

"Some pharmacists had no problems reaching their goal of 10 recruited patients:"

"*“I didn’t really have any trouble getting the 10. They all stuck through the three visits, but a lot of them dropped off for the [post-study] follow-up visit.” (FG3-3)*"

"*“The customers really wanted to be part of the university and they saw it as a privilege.” (FG1-6)*"

Pharmacists reflected on their approach to patient recruitment, suggesting that recruitment is a developmental process, despite their training in how to identify and approach suitable patients:

"*“I could've been a lot more careful about whom I recruited … [later] I found candidates who would’ve benefited more from the program … I thought, ‘oh gosh, I wish I'd made you one of my candidates.’” (FG3-2)*"

"*“I’d probably do it differently if I did it again … I did it by going back through script records to see who would be suitable, and … the ones that I ended up recruiting … probably weren’t the 10 people who would benefit most from that program … I still feel as though they have all benefited from it, but I think I’d put a little bit more thought into how I would pick them out again.” (I2-V4)*"

A number of the pharmacists found themselves recruiting mainly older patients into the service, apparently due to their availability and commitment:

"*“I had around 10 young people who didn’t actually turn up for their appointments … but certainly semi-retired men and women were perfect.” (FG1-7)*"

"*“It was very, very difficult to get working people to come … I had to [consult them] on Saturday afternoons … just a time that they could find to fit me in.” (FG3-2)*"

Once recruited, lack of commitment by patients caused frustration for the pharmacists. Despite reminders, some failed to attend appointments. Patients’ reluctance to persist with the program may have resulted from the long initial consultation, which involved the most research documentation. One pharmacist reflected after the study that the most challenging group to recruit, young males, *“probably needed the program the most”* (FG3-2).

### **Critique of the service components**

The pharmacists largely reflected positively on the practical application of the service. The provision of spirometry was well received by pharmacists, and reportedly, their patients, providing a unique element to the service. Some of the pharmacists would have liked to perform the spirometry earlier in the visit to engage the patient:

"*“They loved looking at the graphs and trying to understand what this meant and what that figure meant … trying to get them to use the spirometer in such a way that they got an ‘A’ quality on their report …They were there to try and beat the machine.” (I2-N7)*"

Criticisms of the service components pertained to the need for more educational aids for patients, a more efficient system to monitor patients’ attainment of their therapeutic goals, and frustration with the amount and flow of the research documentation, which was perceived to deter patient continuance:

"*“[The research documentation] needs to be very careful that it’s not overly clunky, that it’s not too big and unwieldy. Too big and unwieldy will scare people away.” (I2-N6)*"

"*“Every time I see the package lying on my desk, I just look through the paperwork and shake my head, and think … whether it was shown to someone who actually spends their day at the coalface … The way the questionnaires are laid out … they just don’t flow very well … maybe you need a sort of panel … of community pharmacists that you can just call on … for a bit of input before things are being finalised.” (FG3-5)*"

The pharmacists developed a flexible approach in their assessment of individuals’ needs within the bounds of the service:

"*“Some people love it. They’d come in every month if you want them to … they’re those sort of people that … like other people to be looking after them, whereas you get the other ones … you could spend half an hour with them and that would be fine.” (I3)*"

"*“You had to adjust mentally, I suppose, prioritise the information you felt that each particular patient needed … You physically can’t give someone 20 pieces of information.” (FG3-5)*"

### **Logistics of service provision**

The dominant theme relating to logistics of the service was the need for the pharmacist to be available to provide the service. Engaging a locum and/or changing staff rosters may be necessary to deliver the service:

"*“I’ve had to try and get a second pharmacist in and try and schedule the appointments in one after the other on like a certain morning a week. The hardest part is trying to free up myself to do it.” (I2-V3)*"

"*“It was purely time management for me. I had to manage my time personally better, and we had a diary book that we booked people in, because we would use the [private] room for other things.” (FG2-V1)*"

The pharmacists were very conscious of the need to provide undisturbed consultations, which raised further issues around workflow, privacy and location of the consultation.

### **Relationships with GPs**

A feature of the service was the role of the pharmacist as a both a clinical advisor and intermediary between patients and GPs. This required awareness of the service by GPs and communication pathways to propose interventions in individuals’ asthma management. The pharmacists reflected on the need for more formalised and proactive communication strategies with GPs. Pharmacists’ attempts to communicate directly with GPs to discuss the service or therapeutic recommendations for patients were variable, as this is a new type of service in primary care, and no pharmacist reported any GP-initiated communication:

"*“They [GPs] didn’t seem to know anything about it, despite introductory letters obviously being sent out by ourselves and also from you [researchers], so there was a lot of time spent in educating them.” (FG1-3)*"

"*“I collected a whole list of local doctors and sent them letters and tried to get some sort of response, but unfortunately, none of the GPs really care about this.” (FG2-N1)*"

"*“You try and be diplomatic in how you pitch [the clinical recommendation] … from the point of view of saying it’s another idea, and you put all of the dot points down with question marks in front of them, because obviously there may be information that the GPs have that you’re not privy to.” (I2-N5)*"

"*“Most of my communications to [GPs] about plans are written, so there’s really no verbal communication.” (FG2-Q2)*"

The pharmacists perceived that the underlying issues were misunderstanding about the service and the potential threat to GPs’ roles and income stream:

"*“Not so much resentment, but a misunderstanding. ‘Well, why on earth are you doing this sort of thing? Why are you interfering with what seems okay to me?’” (FG3-2)*"

"*“He [the GP] was just concerned that because he hadn’t acted on anything, I was actually going to report him.” (FG2-Q1)*"

Successful interventions mediated by contact with GPs included requests for asthma action plans and medication changes:

"*“I actually suggested changes to medication in probably four or five people. The doctors weren’t at all perturbed about that. They gave them a go.” (FG3-2)*"

"*“I asked [my patients] to take the written notes to the doctor next time they visited, which most of them did.” (FG2-V1)*"

"*“I asked for quite a few asthma action plans, and it’s quite comical the funny little scraps of paper the patients were given … I would’ve hoped for something a little bit more user friendly.” (FG3-1)*"

### **Perceptions of the patients’ experiences**

Overall, feedback from pharmacists about patients’ experience of the asthma service was very positive, translating to enhanced professional pride and relationships with patients:

"*“Just that little bit of interaction they have with you on those visits makes a world of difference. They find you much more approachable.” (FG1-4)*"

For many patients, this comprised a new type of interaction with their pharmacist, and the pharmacists considered themselves ‘change agents’:

"*“One of my guys was just amazed that somebody did want to talk about his asthma.” (FG1-3)*"

"*“I found that middle-aged men … were the least cooperative … least likely to make a good effort on the spirometry and all that kind of thing. But in the end they did, because … they did actually see improvement … they actually became more enthusiastic about the spirometry.” (FG3-2)*"

A common theme, for those patients who engaged with the program, was improved asthma control and improved understanding of the condition. Pharmacists proudly reported positive feedback from their study patients as health improvements became apparent, and this patient feedback was key to the pharmacists’ motivation and professional satisfaction throughout the service delivery:

"*“They hadn’t been to an asthma educator … and they find [the service] really, really useful in terms of understanding inhaler technique and … goal setting.” (FG2-N4)*"

"*“One of the ladies I found quite interesting … through discussion, we found out that she was probably an … ibuprofen-sensitive asthmatic. We’ve got her off it, we’ve got her onto another anti-inflammatory, and she’s been great ever since … so she’s kind of been singing our praises, which is kind of nice.” (I2-V5)*"

The variable nature of asthma control and commitment by patients resulted in some challenges for the pharmacists, suggesting the need for longer-term involvement with at-risk patients:

"*“One of my patients … gave up smoking, and everything improved. Then for the final visit, everything was downhill again.” (FG3-4)*"

"*“Often, you’d do the spirometry and they’d be okay, but … they would then deteriorate and possibly even get better by the time they came and saw you the next time.” (FG3-4)*"

"*“Some of my results I think were a bit skewed because of … dust storms or winter or whatever. Some people with asthma deteriorated, when I would have hoped it would improved as a result of PAMS … as the study progressed, I realised I was probably out of my depth with them.” (FG3-1)*"

### **Professional rewards**

Satisfaction from enhanced clinical practice and empathy were evident in pharmacists’ reflections on their involvement, even early in the program:

"*“I really enjoy these programs because it’s a side of your professional career that you don’t get to ‘do’ very often.” (FG3-5)*"

"*“I suppose it’s really brought asthma into focus in our pharmacy … we are a lot more focused on helping people with their inhaler technique and checking whether devices are appropriate for people.” (FG1-5)*"

"*“I think I’m far more attuned to them [people with asthma] and I think I can communicate well with them because I think I know where they’re coming from.” [FG3-7]*"

There were also changes that reportedly occurred in the pharmacy business as a result of the service, such as patients committing to the pharmacy’s smoking cessation program and a perception of greater client loyalty.

### **The future**

Although there were no clear trends between the three phases of data collection, a number of pharmacists envisaged a future for this asthma management service throughout the trial. Suggestions included extending the service to children. Practical developments such as allocating a day each week for drop-in consultations, and the provision of a mobile out-of-hours asthma service, may address difficulties with issues of patient commitment. Further, reduction in the research-related documentation would shorten the initial consultation and enhance efficiency. The pharmacists’ support was exemplified by:

"*“I would like to continue with it for a lot of reasons. It fulfils a professional need in me, and I like it in that respect. It can be seen by people as a professional service.” (FG2-N3)*"

"*“We’ve got to put into people’s minds that pharmacy can provide other types of services … part of that is all about public relations; part of that is to provide a service and kick it around for a while until we find out what works and what doesn’t work.” (FG2-N3)*"

A concern for the unmet needs of asthma patients founded comments about sustaining the program:

"*“There’s no-one better placed to pick up poorly-controlled asthmatics than pharmacy.” [FG3-6]*"

A future for the program would depend on an established remuneration process for the pharmacists. Pharmacists favoured a patient co-payment system so that consumers would be required to contribute towards the costs of its provision (and therefore associate a monetary value to the service), and yet it would remain affordable to the majority if it were Government-subsidised. An alternative suggestion was to involve other types of practitioners, such as nurse practitioners, in this service.

## Discussion

This paper explores pharmacists’ experiences as service providers in an implementation trial of a novel evidence-based patient-focused specialist asthma service. We have elicited their views on recruitment to the service, how their patients received the service, interactions with other health care professionals, particularly general practitioners, the impact of the service on their role, and their opinions on offering such a service in future. A number of the pharmacists expressed what we perceived would be long-term improvements to their professional services (e.g. developing a habit of checking inhaler technique) or a desire to adopt this as an on-going service. The pharmacists involved in this trial could be considered ‘early adopters’, [14,15] and their engagement with a new style of practice provided insight into challenges and enablers [18] for extended pharmacy services.

Our data were collected predominantly through focus groups, supplemented by individual interviews for the participants’ convenience. The combination of methods balanced the richness of in-depth individual reflections with data gained from group discussion [21]. The interviewers and focus group facilitators were members of the academic research team. While the value of an independent facilitator in feedback studies is recognised [22], the direct involvement of the academic researchers in the data collection ensured familiarity with the protocol by the interviewers, and allowed immediate feedback to be given to the pharmacists to clarify the study protocol.

The pharmacists, on the whole, reported positive experiences with the program for themselves, their pharmacies, and their asthma patients. This is consistent with an Australian trial of pharmacists’ involvement in the prevention of cardiovascular disease, [10] and suggests a future for pharmacists’ engagement in disease state management services. The pharmacists themselves envisaged a future for the service, with some specific needs, such as remuneration and inter-professional communication, to be addressed. The pharmacists’ new-found experience with structured patient consultations and associated documentation should benefit their ongoing practice, regardless of the future of this specific service in their pharmacy.

There are numerous insights from the pharmacists’ reflections on their experience of the asthma service that demonstrate they have been considering how they might continue to implement the service on completion of the study. Some pharmacists expressed regret at the expedient manner in which they recruited patients for the study, and proposed different methods of recruitment that allowed them to target specific patients as they presented. A frequently expressed theme was the desire to expand the service to children. Pharmacists also reflected on the variable nature of asthma and therefore the need for an ongoing service. This, coupled with strongly expressed professional satisfaction, indicated that this group of pharmacists had developed the necessary elements to offer a sustainable asthma service.

The fundamental nature of the service required pharmacists to act as an intermediary between patients and prescribers. A number of pharmacists were challenged clinically by complex patient cases, testament to the need for collaboration between pharmacists and GPs for such patients. The extent of this collaboration, however, was often less than ideal, consistent with research conducted into pharmacist-GP relationships in Australia, [23] which reported a “generally favourable attitude towards one another” but “limited understanding/confidence in the breadth of knowledge of their cross-disciplinary colleagues” and Canada, in which GPs was perceived as “gatekeepers controlling patients’ access to pharmacy services that are unrelated to dispensing [18]”. Pharmacists in our study also reported variable commitment to the program by some patients (working people and younger patients), presumed due to undervaluing of the service by these patients, inability for the organisation and delivery of the service to meet their needs, and/or the lengthy consultations associated with research documentation. Despite this, numerous comments reflected significant professional satisfaction gained through enhanced patient interaction and improved health outcomes. As in an earlier study of inhaler technique education, [24] these positive experiences led some pharmacists to apply skills and tools acquired during the study to non-study patients.

The pharmacists were required to adopt a research culture and to commit to being pioneers of a new professional initiative. Few, if any, of the pharmacists would have received formal university training on research or evidence-based practice, and their involvement in this program was an opportunity for them to provide a structured service independently, yet under guidance of academic researchers. The ‘hand holding’ was a feature of the study design, as it was expected that engagement of the pharmacists with the academic researchers would be key to their commitment to this project. It should be noted that the nature of the researchers’ support was operational, without clinical judgement or input into the patient cases managed by the pharmacists. The training and credentialing ensured that the pharmacists were individually clinically competent, and were empowered to continue providing this service following the conclusion of the study.

Pharmacists’ comments regarding spirometry as a highlight of the training day, and requesting more training in spirometry measurement and interpretation, likely reflect the extension of their knowledge and skills in a related but normally unfamiliar area. This, coupled with their reflections on participants’ interest in exploring their own spirometry results, demonstrate the ability of spirometry to facilitate engagement between patients and health practitioners on asthma and other respiratory disease management. The inclusion of spirometry (or indeed, any diagnostic procedure requiring the pharmacist’s involvement) limits this type of service to face-to-face consultations, which further engages the patient in a direct care relationship with the health practitioner. The low rate (9 %) of spirometry measurement at general practice encounters for the management of asthma in Australia [25], despite annual measurement being recommended, indicates the importance of expanding the range of health professionals who are competent at spirometry measurement, with pharmacists being ideally placed to offer this service. Future iterations of this asthma management service could focus more on the fluctuations of asthma control, and the visits could be tailored accordingly. Pharmacists must be cognisant of how fluctuations appear in spirometry measurements and the level of asthma control, to be expected to respond with appropriate medication.

One limitation in our approach is that, despite the use of three time points for pharmacists’ feedback, we did not longitudinally monitor each pharmacist’s experiences. Further, the lack of commitment or availability of younger patients and greater enthusiasm of older patients, as reported by a number of pharmacists, may have influenced the pharmacists’ experiences with this trial. Finally, the pharmacists may have felt “obliged” to give positive feedback to the facilitator. To minimise this response bias, for the focus groups, one member of the team whom they had not met in training and had not visited them at their pharmacy was the facilitator. We found that the elucidation of ambivalent and negative experiences (“disconfirming evidence”) [26], particularly regarding the study workload, suggested that the focus groups did not foster ‘normalisation’ or tempering of the pharmacists’ comments for the researchers, and that the discussions were facilitated in a supportive environment. Similar experiences were reported by researchers in trials of diabetes management services in Australia. [8,13]

We have derived a number of recommendations for optimal involvement of pharmacists in a multi-centre academic research trial of this nature, and therefore to inform wider implementation of this asthma service and design of trials for other services:

· The pharmacists should be provided comprehensive training in their areas of need (e.g. skills in spirometry, communication with GPs), with credentialing to ensure competence.

· The training component of the service should involve training in the research requirements (specifically patient recruitment, consultation and documentation) and the physical requirements for provision of professional services (work flow, staffing, appointment systems and the consultation area).

· Research-related paperwork and data collection should be minimised.

· The researchers should provide support in the communication and feedback with/by the investigators, and via site visits, to enhance the pharmacists’ confidence and engagement; the interaction should be managed carefully to preserve the relationship between the pharmacists and academic researchers.

· Balance is required between imposing on the pharmacists a structured research protocol, providing operational support for their involvement, and affording them the flexibility to manage individual patients.

· The researchers should acknowledge that despite the pharmacists’ commitment to research, the core businesses of community pharmacy will be their priority.

· The opportunity for involvement in research can be promoted to pharmacists as a business model in terms of investments (training, workplace support) and returns (improved patient outcomes and loyalty).

Pharmacists’ involvement in an academically-managed trial of a new service is likely to have benefits reaching more broadly than to the select patient cohort under study. Further, it is hoped that by engaging with the pharmacists as research partners, and instilling a research culture into community pharmacy practice, this study has generated goodwill for future pharmacy-based research initiatives.

## Conclusions

The feedback from the study pharmacists was positive overall, yet constructively critical of some aspects of the research protocol, which was not surprising considering the volume of research data being collected. The pharmacists were engaged as research partners, recognising their role as pioneers of a novel, clinically advanced service model for other Australian pharmacists. They understood the need for the service and were able to give valuable feedback about its future. Key challenges for the pharmacists related to recruitment of asthma patients, time management, and collaboration with GPs. Overall, their positive experiences demonstrated that if the challenges were managed strategically, implementation of such a service model would be possible.

## Competing interests

The authors declare that they have no competing interests.

## Authors’ contributions

All authors contributed to the study design, the preparation of clinical materials, interpretation of data and writing of this manuscript. LS and CA were involved in conducting the focus groups. KL and CA performed the initial data synthesis from the focus groups. LE performed the detailed data analysis and drafting of the manuscript. All authors have read and approved the final manuscript.

## Pre-publication history

The pre-publication history for this paper can be accessed here:

http://www.biomedcentral.com/1472-6963/12/164/prepub

## Supplementary Material

Additional file 1 Interview guide (truncated to remove introductory statements and prompts). Click here for file
