# Supplementary material for: Experiences of community pharmacists involved in the delivery of a specialist asthma service in Australia
Source: BMC Health Serv Res. 2012 Jun 18;12:164. doi: 10.1186/1472-6963-12-164 (PMC3439711; doi:10.1186/1472-6963-12-164)
Supplement: Additional file 1 — Interview guide (truncated to remove introductory statements and prompts). [file 1472-6963-12-164-S1.docx]

# Additional files

### Additional file 1 – Interview guide (truncated to remove introductory statements and prompts)

*Pharmacist’s experience*

- What has been your overall experience with PAMS so far?
- How well did the training equip you to deliver the service? What worked particularly well/helped to prepare you? Were there any gaps in the training?
- What has been your experience in recruiting patients?
- How easy or difficult has it been to implement the PAMS service in your pharmacy?
- What did you think of the service protocol now that you have tried it?
- What was the average time you spent with a patient during a PAMS visit?
- Did you provide 3 or 4 visits per patient over the 6-month service? Did you find that it was enough visits with your patients? Would you have preferred more or fewer visits?

*Pharmacist’s perspective of patient’s experience*

- Thinking about the service from your patients’ perspective, how do you think the service has been received by your patients so far?
- What have been the most useful parts of the service (so far) for your patients? Least useful parts of the service?
- How do you think the service could be improved for patients in the future?

*GP/Specialist interaction with the service*

- Have you had contact with GPs as a direct result of your involvement with PAMS? If so, in what ways?
- Did you refer patients to the GP, or did GPs refer patients to you?
- What happened after the referral, if anything?
- Are you aware if any GPs initiated asthma services for your PAMS patients during the service?
- What, if any, impact has your involvement with PAMS had on your professional relationship with local GPs?
- Overall, what do you think that GPs thought of the PAMS service?

*Other impacts*

- What other spinoffs, if any, have you experienced so far as a result of offering the service?

*The Future*

- How much demand do you think there is for a service like this in community pharmacy?
- How do you think this service could be improved in the future?
